# Consumers’ perceptions of energy use and energy savings: A literature review

**DOI:** 10.1088/1748-9326/aaab92

**Published:** 2018-03-06

**Authors:** Vedran Lesic, Wändi Bruine de Bruin, Matthew C Davis, Tamar Krishnamurti, Inês M L Azevedo

**Affiliations:** 1 Centre for Decision Research, Leeds University Business School, University of Leeds, Leeds, LS2 9JT, United Kingdom; 2 Department of Engineering and Public Policy, Carnegie Mellon University, Pittsburgh, PA 15213, United States of America; 3 Socio-Technical Centre,Leeds University Business School, Leeds, LS2 9JT, United Kingdom; 4 Author to whom any correspondence should be addressed.

**Keywords:** perceptions of energy consumption, actual energy use, smart meters, savings potential, residential sector

## Abstract

**Background.:**

Policy makers and program managers need to better understand consumers’ perceptions of their energy use and savings to design effective strategies for promoting energy savings.

**Methods.:**

We reviewed 14 studies from the emerging interdisciplinary literature examining consumers’ perceptions electricity use by specific appliances, and potential savings.

**Results.:**

We find that: (1) electricity use is often overestimated for low-energy consuming appliances, and underestimated for high-energy consuming appliances; (2) curtailment strategies are typically preferred over energy efficiency strategies; (3) consumers lack information about how much electricity can be saved through specific strategies; (4) consumers use heuristics for assessing the electricity use of specific appliances, with some indication that more accurate judgments are made among consumers with higher numeracy and stronger pro-environmental attitudes. However, design differences between studies, such as variations in reference points, reporting units and assessed time periods, may affect consumers’ reported perceptions. Moreover, studies differ with regard to whether accuracy of perceptions was evaluated through comparisons with general estimates of actual use, self-reported use, household-level meter readings, or real-time smart meter readings.

**Conclusion.:**

Although emerging findings are promising, systematic variations in the measurement of perceived and actual electricity use are potential cause for concern. We propose avenues for future research, so as to better understand, and possibly inform, consumers’ perceptions of their electricity use. Ultimately, this literature will have implications for the design of effective electricity feedback for consumers, and related policies.

## 1. Introduction

The use of fossil fuels in electricity generation is one of the major contributors to greenhouse gas emissions (GHG) worldwide (Intergovernmental Panel on Climate Change 2014). A large de-carbonization of the energy system is necessary to reduce and stabilize carbon dioxide (CO_2_) and other GHG emissions in the atmosphere (IPCC 2014). A portfolio of de-carbonization strategies and technologies will likely include curtailment (which is also called ‘energy conservation’ in much of the energy literature) and energy efficiency strategies targeting the reduction of residential energy use (IPCC 2014, Pacala and Socolow 2004). Curtailment strategies and pertain to actions consumers can pursue to reduce the energy use of existing appliances by using them less or not at all (Azevedo 2014, Rubin *et al* 1992). Energy efficiency strategies involve the implementation of more efficient appliances (Karlin *et al* 2014). If people misjudge the relative energy use or savings of one appliance or action over another, their efforts to save electricity may end up being misdirected.

Consumers with more accurate perceptions of energy use and savings may be better able to identify the actions that save the most energy, as a first potential step towards behavior change and reduced GHG emissions. Providing consumers with better information about their energy use and potential savings brings the promise of promoting the implementation of more curtailment and energy efficiency strategies and reducing residential greenhouse gas emissions (Bin and Dowlatabadi 2005, Vassileva *et al* 2012, Attari *et al* 2010, Attari 2014, Baird and Brier 1981, Chen *et al* 2015, Frederick *et al* 2011, Kempton and Montgomery 1982, Mettler-Meibom and Wichmann 1982, Schley and DeKay 2015). Many consumers want better information, and hope that smart meters will help them to understand how much electricity is used by specific appliances (Krishnamurti *et al* 2012). Without information, consumers may develop folk theories and associated misconceptions about their energy use (Kempton 1986, Kempton and Montgomery 1982, Krishnamurti *et al* 2013).

This paper aims to understand how well consumers can assess the electricity used by different household appliances, and how much can be saved by implementing different curtailment or energy efficiency strategies. We provide a systematic overview of the empirical studies that have focused on the accuracy of consumers’ perceptions of energy consumption and energy savings for specific appliances and actions. The paper is organized as follows. First, we briefly describe how we selected the studies that are included in this paper. Second, we discuss the key empirical findings reported in these studies. Third, we describe methodological differences in terms of how studies have measured consumers’ perceptions of energy use. Fourth, we discuss the different ways in which actual energy consumption has been measured across studies, so as to evaluate the accuracy of consumers’ perceptions. Finally, we conclude with recommendations for future studies and implications for developing effective feedback design and programs.

## 2. Methods and data

We performed a search for studies that used all possible combinations of the following keywords: ‘consumer perceptions’, ‘consumer awareness’, ‘energy consumption’, ‘energy use’, and ‘energy savings’. We searched the following online databases: ScienceDirect, EBSCO, general library catalogues of Carnegie Mellon University and University of Leeds, limiting our search to articles published after 1980. From this initial search, we only retained peer-reviewed articles that reported the direct results of experimental, survey, or interview research with human participants. We also searched for studies in Google Scholar (where we focused solely on the first 25 pages of results). We read the abstract of each of the papers (and when it was unclear from the abstract, we also read the full paper to assess if a study would remain in our final dataset). We focused on identifying the papers that specifically reported perceptions or awareness of energy use and savings. Our initial search identified 32 peer-reviewed papers. We also identified six additional peer-reviewed papers in the references of these 32 papers. We included one additional paper on the basis of a reviewer’s recommendation. In appendix table A1 we present the resulting 39 papers. We then read each of the 39 papers to identify those papers that met the inclusion criteria of: (1) focusing…. (2) presenting and (3) measuring actual use without necessarily making a comparison of actual use with perceptions (see table 1). Our review covers the resulting 14 studies that meet the inclusion criteria. For example, Allcott’s (2011) paper on fuel energy consumption or Becken’s (2013) paper on perceptions of energy use and actual saving opportunities for tourism accommodation made it into the initial selection of 32 papers but did not made it to final review because they are not in the domain of residential energy use. Of the 14 studies we reviewed, ten papers specifically presented comparisons of assessed perceptions and actual use (see table 1).

## 3. Main empirical findings

We identify four main empirical findings across the 14 studies in our review:

1. Consumers have systematic misperceptions of energy use, such that electricity use is often overestimated for low-energy consuming appliances, and underestimated for high-energy consuming appliances (Attari *et al* 2010, Baird and Brier 1981, Chen *et al* 2015, Frederick *et al* 2011, Gatersleben *et al* 2002, Kempton and Montgomery 1982, Mettler-Meibom and Wichmann 1982, Schley and DeKay 2015);
2. Consumers tend to prefer curtailment over energy efficiency strategies (Attari *et al* 2010, Becker *et al* 1979, Kempton *et al* 1985, Mettler-Meibom and Wichmann 1982);
3. Consumers lack information about the electricity savings associated with specific strategies (Attari *et al* 2010, Easton and Smith 2010);
4. Consumers use heuristics for assessing the electricity use of specific appliances (Baird and Brier 1981, Schley and DeKay 2015), with some indication that more accurate judgments are made among consumers with higher numeracy and stronger pro-environmental attitudes (Attari *et al* 2010, Schley and DeKay 2015).

We discuss each of these findings in turn in the sections below.

### 3.1. Systematic misperceptions of energy use

Consumers tend to systematically overestimate the electricity use of low-energy consuming appliances and activities, while underestimating the electricity use of high-energy consuming appliances and activities (Attari *et al* 2010, Chen *et al* 2015, Frederick *et al* 2011, Gatersleben *et al* 2002, Kempton and Montgomery 1982, Mettler-Meibom and Wichmann 1982, Schley and DeKay 2015). In one study, participants reported their perceived energy use for nine appliances, in terms of their hourly electricity use in kWh (Attari *et al* 2010). Participants received a reference point of a 100 W incandescent light bulb when making their assessments. The accuracy of perceptions was evaluated by comparing perceptions to actual energy use, as estimated from the literature and government agencies. According to the authors, participants underestimated the energy use of the nine appliances by a factor of 2.8 on average, while also overestimating the electricity use of low-energy consuming appliances (Attari *et al* 2010). A follow-up study asked participants to consider the same nine appliances, while providing either a 3 W LED, a 100 W incandescent light bulb or a 9000 W electric furnace as the single reference point (Frederick *et al* 2011). Frederick *et al* (2011) used the same estimates for actual energy use and savings as Attari *et al* (2010). Participants reported higher perceptions of electricity use across the nine appliances when they were presented with a higher rather than a lower reference point, with perceptions being highest when no reference point was provided at all (Frederick *et al* 2011). Moreover, overestimations were larger when questions were asked in terms of kWh versus Wh (Frederick *et al* 2011). Although Frederick *et al* (2011) found that the findings of Attari *et al* (2010) depended on reference points and reporting units, the overall pattern of underestimating the electricity use for high-consuming appliances and overestimating it for low-consuming appliances remained (Attari *et al* 2011).

Other studies revealed that same pattern (Chen *et al* 2015, Gatersleben *et al* 2002, Kempton and Montgomery 1982, Mettler-Meibom and Wichmann 1982, Schley and DeKay 2015) despite measuring perceptions and actual use in different ways (table 1) and varying reference points and reporting units (table 2). Regression towards the mean may have contributed to electricity use being overestimated for low-energy consuming appliances and underestimated for high-energy consuming appliances, because perceptions and actual use are imperfectly correlated (Attari *et al* (2010). However, regression towards the mean does not ‘explain’ why the correlation is imperfect, or why reported perceptions depend on how they are assessed. Similar patterns of findings have also been reported with regards fuel consumption (Allcott 2011, Larrick and Soll 2008) and water use (Attari 2014).

### 3.2. Tendency to prefer curtailment strategies over energy efficiency strategies

Several studies in the literature note that consumers tend to choose curtailment strategies over energy efficiency strategies, even though the latter are potentially more effective for saving energy (Attari *et al* 2010, Becker *et al* 1979, Kempton *et al* 1985, Mettler-Meibom and Wichmann 1982). For example, open-ended interviews with Michigan residents revealed that they tended to talk more about curtailment actions such as turning off the lights and lowering the winter thermostat, rather than on energy efficiency actions, such as better house insulation (Kempton *et al* 1985). A similar pattern was found in other open-ended interviews (Mettler-Meibom and Wichmann 1982) and in a national survey that asked participants for strategies to reduce energy use (Attari *et al* 2010). Another study found that most participants overestimated the savings that could be derived from curtailment by lowering the thermostat, as compared to implementing more energy-efficient devices (Becker *et al* 1979). Possible reasons for this preference for curtailment over energy efficiency are (i) that that curtailment is likely to have no financial costs in most circumstances, whereas efficiency will likely involve some form of investment or additional financial cost, e.g. investment in insulation or LED lighting; (ii) curtailment behaviors come to mind more easily than energy efficiency strategies, due to the former being implemented more frequently than the latter.

### 3.3. Lack of information about energy savings

In the absence of information, consumers may use their own experience to create folk theories about how different appliances or behaviors might consume or save energy (Kempton 1986, Kempton and Montgomery 1982). Perhaps as a result, consumers misjudge how much electricity is used by specific appliances and behaviors (Attari *et al* 2010, Easton and Smith 2010). The same pattern of misperceptions is seen in perceptions of energy use and energy savings (Attari *et al* 2010). Indeed, participants tend to overestimate low-consuming actions and underestimate high-consuming ones (Attari *et al* 2010).

Easton and Smith (2010) asked questions related to consumers’ perceptions of energy consumption, energy-related behavior, and energy savings over a year, and then combined the responses to those questions with direct monitoring of metered energy, water, and temperatures provided by four community based retrofit organizations. Notably, they show that households underestimate the extent of repairs and maintenance that is required on their dwellings to save energy.

### 3.4. Heuristics and individual differences

When reporting their perceptions, participants also seemed to use heuristics or decision rules to simplify the task at hand (Tversky and Kahneman 1974). The commonly used ‘availability heuristic’ reflects the tendency to judge the likelihood of an event by the ease with which an example comes to mind (Schwarz *et al* 1991). Individuals who use the availability heuristic tend to systematically overestimate events that come to mind more easily, and underestimate events that come to mind less easily (Tversky and Kahneman 1973). Consumers may also use such heuristics when generating strategies for saving energy (Wilson and Dowlatabadi 2007) and assessing the electricity use of their appliances (Baird and Brier 1981, Schley and DeKay 2015). Specifically, participants judge electricity use to be higher for appliances that are frequently used or thought of (Schley and DeKay 2015) as well as those that are larger in size (Baird and Brier 1981). Such heuristics will lead to predictable inaccuracies, such as for infrequently used appliances that use relatively more electricity or frequently used appliances that use relatively little (Baird and Brier 1981). Similarly, curtailment actions may come to mind more easily than energy-efficiency actions due to being implemented more frequently—leading to overestimations of the associated energy savings.

Moreover, the accuracy of perceptions may systematically vary across participants. Two studies find that more numerate participants have more accurate perceptions of energy use for specific appliances (Attari *et al* 2010, Schley and DeKay 2015). One study reports that participants with stronger pro-environmental attitudes have more accurate perceptions of energy use and potential savings (Attari *et al* 2010), while another reports that they do not (Schley and DeKay 2015).

## 4. Methodological differences between studies

The studies we reviewed differ in their research method, including qualitative interviews (Easton and Smith 2010, Kempton and Montgomery 1982, Mettler-Meibom and Wichmann 1982), and surveys (Abrahamse *et al* 2007, Abrahamse and Steg 2009, Becker *et al* 1979, Gatersleben *et al* 2002, Kempton *et al* 1985, Attari *et al* 2010, Baird and Brier 1981, Chen *et al* 2015, Frederick *et al* 2011). Across these research methods, we identify three methodological features that may affect consumers’ reported perceptions of electricity use:

- the presence or absence of a reference point, with reference points varying in size from a 3 W LED (Frederick *et al* 2011), to a 100 W incandescent light bulb (Attari *et al* 2010, Frederick *et al* 2011), and even a 9000 W electric furnace (Frederick *et al* 2011);
- the units in which consumers report their perceptions of electricity use, such as in kWh (Attari *et al* 2010, Baird and Brier 1981) or in dollars (Karjalainen 2011);
- the time periods in which consumers report their perceptionsof electricity use, suchasperhour (Attari *et al* 2010, Baird and Brier 1981, Frederick *et al* 2011), per month (e.g. Mettler-Meibom and Wichmann 1982) or per year (Easton and Smith 2010: Schley and DeKay 2015).

### 4.1. Reference point

Behavioral decision researchers have long suggested that the provision of a reference point, or comparison information, affects people’s reported perceptions (Hammond *et al* 1998, Sunstein 2002). That is, people tend to adjust their perceptions towards the reference point that is provided (Chapman and Johnson 2002, Attari *et al* 2010). Some studies in our review provided reference points to participants with the aim of helping them generate their perceptions (table 2). For example, studies have presented information about the electricity use of a 3 W LED (Frederick *et al* 2011), a 100 W incandescent light bulb (Attari *et al* 2010, Frederick *et al* 2011), a 100 W washing machine (Baird and Brier 1981), and a 9000 W electric furnace (Frederick *et al* 2011). Perhaps not surprisingly, participants report higher perceptions of electricity use when being presented with a higher rather than a lower reference point, with perceptions being highest when no reference point is provided at all (Frederick *et al* 2011). Future studies should test whether the provision of multiple reference points provides information about the feasible range, without biasing judgments upwards or downwards, as compared to when no reference point is provided.

### 4.2. Reporting unit

Some studies asked participants to report the electricity use of their appliances in different units of consumption (table 2), such as kWh (Attari *et al* 2010, Baird and Brier 1981) or dollars (Becker *et al* 1979, Easton and Smith 2010). When describing the energy consumption associated with their home heating, most people tend to refer to monetary values (Kempton and Montgomery 1982). Indeed, consumers may be more familiar with monetary units than with energy units because of the salience of paying electricity or heating fuel bills (Darby 2006). As a result, they may want to see feedback about their electricity use displayed in terms of monetary units rather than energy units (Karjalainen 2011). However, simple feedback provided in energy units may be the most effective way to increase knowledge about energy use (Krishnamurti *et al* 2013). Behavioral decision studies in other domains suggest that consumers may overestimate prices as compared to other units (Bruine de Bruin *et al* 2011, Vohs *et al* 2006). Because of the small sample sizes and variability in study designs, it is unclear at this stage whether monetary units or energy units might be better at helping consumers to judge their electricity use. Future research should systematically test the effect of reporting units on consumers’ perceptions of how much electricity is used by their appliances.

### 4.3. Time period

Studies vary in terms of the time period participants have considered when reporting their perceptions of appliance’s electricity use (table 2). For example, participants have been asked to assess how much electricity an appliance uses over the course of an hour (Attari *et al* 2010, Frederick *et al* 2011), a month (e.g. Mettler-Meibom and Wichmann 1982), or a year (Easton and Smith 2010, Schley and DeKay 2015). The time period may also be left unspecified (Chen *et al* 2015). One drawback of asking consumers about their perceived energy use over the course of an hour is that comparisons with actual use may not be realistic (i.e. it may not make sense to ask how much energy a coffee machine or a toaster uses if it is running for a full hour, sincethatdoesnotreflectusualusagepatterns). Instead, the researcher may ask participants for the frequency of use of an appliance and the energy use over that period. Additionally, the time period consumers are asked to consider may affect their reported perceptions. Monthly periods may be more familiar to people given that historically most utilities would send monthly utility bills. Yet, technology that enables consumers to receive more frequent electricity use information is available (Anderson and White 2009) and some work has shown that consumers are interested in seeing information such as daily load curves (Ueno *et al* 2006). In other research that does not focus on energy use, researchers have found that self-reported hours of TV watching depend on the time period used in the survey, with more accurate responses being provided when time periods match people’s natural experiences (Schwarz 1999).

Although none of the reviewed studies examined whether assessed time periods used affects perceptions, there is reason to believe that they might. Especially when considering longer time periods, participants may assume the appliance is running for the full duration of that time period, or they may assume what is a ‘typical’ usage of the appliance for them. If participants make different assumptions about how to respond to such questions as the time period increases, their reported perceptions will likely show a larger variability. If perceptions are to be reported for typical use over a time period, it is important to note that people often misestimate the amount of time they spend on tasks (Fasolo *et al* 2009). They may overestimate the electricity use of appliances they tend to use longer (Yeung and Soman 2007). In addition, behavioral economics research on magnitude effects suggests that people display a larger subjective temporal discount rate for small magnitudes than for large ones (Chapman and Winquist 1998). Thus, it may be easier to think of specific appliances in terms of their relative time periods of use.

## 5. Measures of actual energy use

This section focuses on the methods for measuring actual energy use and energy savings, so as to assess the accuracy of consumers’ reported perceptions. The 14 studies identified in our review that include a measure of actual energy use can be divided into four categories with regards how they measured actual energy use:

1. *General estimates from the existing literature and other sources* (these include Attari *et al* 2010, Becker *et al* 1979, Baird and Brier 1981, Frederick *et al* 2011, Mettler-Meibom and Wichmann 1982, Kempton *et al* 1985, Schley and DeKay 2015);
2. *Estimates based on self-reported energy use* (these include Gatersleben *et al* 2002, Abrahamse *et al* 2007, Abrahamse and Steg 2009);
3. *Estimates based on household-level meter readings* (thisincludesKemptonandMontgomery1982,Easton and Smith 2010);
4. *Measures of real-time energy usage from smart meters* (Chen *et al* 2015).

Each of these approaches has its own set of advantages and disadvantages, as summarized in table 3. In table 3, we provide our assessment of these four approaches on five criteria, on a scale ranging from very low to very high: (1) data accessibility, which refers to the ease of obtaining the data, (2) cost of measurement, which refers to how costly it might be to gather the data, (3) data accuracy, which refers to the extent to which the data reflect actual energy consumption rather than an estimate, (4) data complexity, which refers to the level of analysis needed to prepare, store, and compute the data, and (5) third-party involvement, which refers to the need to involve other organizations in obtaining the data.

### 5.1. General estimates from the existing literature and other sources

Many of the reviewed studies used general estimates of energy use or energy savings of specific appliances and behaviors, so as to evaluate the accuracy of participants’ reported perceptions (table 1). Some studies used publicly available estimates from existing publications including expert reports (Becker *et al* 1979, Mettler-MeibomandWichmann1982, Kempton *et al* 1985), energy statistics from for example governmental agencies (Attari *et al* 2010, Frederick *et al* 2011, Schley and DeKay 2015), or information from local stores (Baird and Brier 1981). Using these sources is convenient because they are readily available. However, this approach comes with the severe limitation of not capturing individual heterogeneity in consumption. As a result, it is impossible to know whether any differences between perceived and actual consumption are due to misperceptions by the consumer or due to average energy use being a poor proxy for the actual energy consumption of a specific household.

### 5.2. Estimates based on self-reported energy use

It is also possible to estimate an individual’s actual energy use for specific appliances from self-reports (Abrahamse *et al* 2007, Abrahamse and Steg 2009, Gatersleben *et al* 2002). Gatersleben *et al* (2002) developed a model to calculate actual energy consumption based on participants’ self-reported behavior. The authors asked participants to report which appliances they own. For each appliance, the total number of appliances of that type in the household was multiplied by the average annual energy use of the appliance as estimated for an average Dutch household.

Estimates of actual energy use by appliance were then computed for individual participants and compared to their reported perceptions of energy use. The benefit of this approach is that individuals’ perceptions are compared to their own usage patterns and appliances. However, one limitation is that participants may not know the required information, or provide inaccurate reports due to imperfect memory or response biases (Baumeister *et al* 2007). Another drawback of self-reports is that they may be labor-intensive for participants to complete, especially if the study includes a large number of appliances.

### 5.3. Estimates based on household-level meter readings

Another approach is to estimate an individual’s energy use for specific appliances after obtaining a household-level meter reading from the utility company. Since the late 1970s, many studies have evaluated the accuracy of consumers’ perceptions of electricity, gas, or water use on the basis of meter readings provided by utility companies (e.g. Heberlein and Warriner 1983, Hirst *et al* 1982, Kempton and Montgomery 1982, Midden *et al* 1983, Seligman *et al* 1978, Verhallen and van Raaij 1981). The benefit of this approach is that it provides household-specific information, allowing comparisons of individuals’ perceptions with their own electricity use (Schley and DeKay 2015). Various intervention studies (Battalio *et al* 1979, King 2010, Kline 2007) have also used household-level energy data to provide feedback to households and to test the resulting effects on residential energy use. However, household-level readings too come with potential limitations. First, they do not provide information regarding the energy consumption of specific appliances. Second, many studies have relied on monthly assessments from utilities which only conduct actual meter readings a few times per year, and make estimates for the rest of the year.

### 5.4. Measures of actual energyusefromsmart meters

The deployment of smart meters has enabled the measurement of households’ real-time energy consumption (Asensio and Delmas 2015, Chen *et al* 2015). These measurements may include (i) single load monitoring combined with algorithms to estimate the consumption of different appliances, or (ii) multi-modal sensing. Single-load monitoring through smart meters is a non-intrusive method for measuring real-time household-level electricity use and can be combined with specifically designed algorithms to identify when specific appliances are being used (Berges *et al* 2008). Even with advanced algorithms, this approach will involve underlying uncertainty. Instead, multi-modal sensing overcomes that uncertainty through the installation of special sub-meters to capture usage for each appliance (Froehlich *et al* 2011). Sub-meter data facilitate direct comparisons between consumers’ perceived and actual use of appliance-level energy use. Using sub-meter data also allows for better testsof theeffectivenessof interventions. Thisapproach has been implemented in the Pecan Street community located at the University of Austin in Texas (Pecan Street 2017, Smith 2009). However, sub-meters are more intrusive and costly to implement, limiting the feasibility of using them with a large or nationally representative sample.

## 6. Conclusions and recommendations for future studies

Our review of the literature covers 14 peer-reviewed studies that empirically assessed consumer perceptions of electricity use that has been published over the past 35 years. An even smaller number of studies (N=10) compared consumers’ perceptions to actual energy use or savings. The main findings from the reviewed studies include: (1) electricity use is typically overestimated for low-energy consuming appliances, and underestimated for high-energy consuming appliances; (2) curtailment strategies are typically preferred over energy efficiency strategies; (3) consumers lack information about how much electricity can be saved throughspecificstrategies; (4) consumersuseheuristics for assessing the electricity use of specific appliances, with some indication that more accurate judgments are made among consumers with higher numeracy and stronger pro-environmental attitudes.

However, we note that methodological differences between studies may affect consumers’ reported perceptions, including the provision of reference points, as well as the units and time periods used in the existing studies. Moreover, studies vary in terms of whether the accuracy of perceptions has been evaluated in terms of general estimates of actual use, self-reported use, house-level meter readings, or real-time smart meter readings.

We suggest several avenues for future research. First, there is a need to systematically examine the effect of reference points, units, and time periods on reported perceptions. Second, to better compare consumers’ perceptions to their actual appliance energy use, measures of households’ actual energy consumption should be taken at the individual households’ appliance level. Ideally, such studies would be conducted with large representative samples. Moreover, it remains unclear whether consumers with more accurate perceptions of their energy use by appliance, or of the savings they could obtain, do indeed make more informed decisions about their energy use and savings. It also remains to be seen whether informed decisions lead to behavior change and reductions of residential GHG emissions.

Understanding consumers’ perceptions (and misperceptions) of energy use and savings may help to inform the design of curtailment and energy efficiency policies. The use of smart technology and associated services, such as in-home displays, mobile apps, and other information and communication technology related services could facilitate improved measurement as well as improved feedback to consumers (Krishnamurti *et al* 2012). However, care should be taken to present feedback in a way that consumers can use and understand (Davis *et al* 2014). For example, tailored feedback may be provided to consumers to explain their misperceptions, while using reference points, units, and time periods that make the most sense to them. Research should also be developed to then test whether correcting misperceptions through feedback does indeed help consumers to make more informed decisions about curtailment and energy efficiency. In the domain of health, researchers have shown that correcting misperceptions of risk can foster behavior change (Avis *et al* 1989, Kreuter and Strecher 1995, Lindan *et al* 1991). Thus, continued research on the topic of how well consumers can assess appliance energy use brings some promise of informing consumers’ decisions to implement curtailment and energy efficiency behaviors.

## Figures and Tables

**Table 1.** Summary of the studies reviewed.

Author, Year	Method for measuring perceptions	N	Specific measure of perceptions	Includes perceived energy use	Includes perceived energy saving	Specific measure of actual energy use or savings	Compares perceptions with energy use and/or savings
1. Abrahamse *et al* (2007)	Online survey	189	Participants reported perceptions of the energy that could be saved for 27 energy-related behaviors.	No	Yes	Estimated from participants’ self-reported use of appliances and behaviors.	No
2. Abrahamse and Steg(2009)	Online survey	314	Participants reported perceptions of direct and indirect energy use of their household, their current use of appliances and energy-related behaviors.	Yes	No	Estimated from participants’ self-reported use of appliances and behaviors.	No
3. Attari *et al* (2010)	Online survey	505	Participants reported perceptions of the energy used by nine appliances and the energy saved by six energy-related activities.	Yes	Yes	Estimated from existing literature.	Yes. Correlations and regressions were used to assess the relationship between perceptions and estimates of actual energy use and savings.
4. Baird and Brier (1981)	Laboratory experiments	Study 1: 48; Study 2: 24; Study 3: 20	Participants reported perceptions of energy use for 19 appliances.	Yes	No	Estimated from the power reported on labels of the devices in a local hardware store.	Yes. Correlations were computed between perceptions and estimates of actual energy use.
5. Becker *et al* (1979)	Mail survey	43	Participants reported perceptions of energy savings for two winter thermostat settings.	No	Yes	Estimated from existing literature.	Yes. Comparisons were made between perceptions and estimates of actual energy savings.
6. Chen *et al* (2015)	Survey	137	Participants reported perceptions of energy use for three categories of appliances.	Yes	Yes	Estimated from smart meter data in combination with high-frequency load monitoring to disaggregated electricity on appliance-level over 24 months.	Yes. Comparisons were made between perceptions and estimates of actual energy use and savings.
7. Easton and Smith (2010)	Phone interview	232	Participants reported perceptions of energy performance and potential savings for total household.	No	Yes	Measured through direct monitoring of metered energy, water and temperature provided by four communities based retrofit organizations.	Yes. Comparisons were made between perceptions and measurements of actual energy savings.
8. Frederick *et al* (2011)	Online survey	Study 1: 104 Study 2: 77	Study 1 and 2: Same as in Attari *et al* (2010).	Yes	No	Estimated from existing literature.	Yes. Correlations were computed between perceptions and estimates of actual energy use.
9. Gatersleben *et al* (2002)	Mail survey	Study 1:2167; Study 2: 1250	Participants reported perceptions of how harmful eight of their actions are compared to other Dutch households.	Yes	No	Estimated from participants’ self-reported use of appliances, and the average energy use by Dutch households.	Yes. Correlations were computed between perceptions of the environmental impact and their energy use.
10. Kempton and Montgomery(1982)	Ethnographic interview	30	Participants reported perceptions of how they disaggregate their monthly energy use and perceptions of their monetary savings	No	Yes	Measured by local utility company and estimated from existing literature.	No.
11. Kempton *et al* (1985)	Mail survey	400	Participants reported perceptions of the savings for 22 different behaviors.	No	Yes	Estimated from existing literature.	Yes. Comparisons were made between perceptions and estimates of energy savings.
12. Kempton (1986)	Face to face interview	1st round: 30 2nd round: 12	Participants reported perceptions of thermal use.	Yes	No	Behavioral records of thermostat settings collected from Princeton University’s Center for Energy and Environmental Studies over 2 year period.	No.
13. Mettler-Meibom and Wichmann (1982)	Face-to-face interviews	52	Participants reported perceptions of the costs of four energy-related activities.	Yes	No	Estimated from existing literature.	Yes. Comparisons were made between perceptions and estimates.
14. Schley and DeKay(2015)	Online survey	734	Across four studies, participants reported perceptions of the percent of total individual and household energy used annually for 11–16 end-use categories.	Yes	No	Estimated from existing literature.	Yes. Comparisons were made between perceptions and estimates.

**Table 2.** Key methodological features across studies.

	Study	Reference point		Time periods			Reporting units		
		Presented	Not presented	Hourly	Monthly	Yearly	Money	Energy	Other
1	Abrahamse *et al* (2007)		Yes		Yes				Yes
2	Abrahamse and Steg (2009)		Yes		Yes				Yes
3	Attari *et al* (2010)	Yes		Yes				Yes	
4	Baird and Brier (1981)	Yes		Yes				Yes	
5	Becker *et al* (1979)		Yes		Yes		Yes		
6	Chen *et al* (2015)		Yes					Yes	
7	Easton and Smith (2010)		Yes			Yes	Yes		
8	Frederick etal (2011)	Yes		Yes				Yes	
9	Gatersleben etal (2002)		Yes		Yes				Yes
10	Kempton and Montgomery (1982)		Yes		Yes			Yes	
11	Kempton etal (1985)		Yes		Yes			Yes	
12	Kempton (1986)		Yes		Yes				Yes
13	Mettler-Meibom and Wichmann (1982)		Yes		Yes			Yes	
14	Schley and DeKay (2015)		Yes			Yes		Yes	

**Table 3.** Approaches to measure actual energy use.

		Data accessibility	Cost of measurement	Data accuracy	Data Complexity	Third parties data needed
1	General estimates from the existing literature and other sources	**Very high**	**Very low**	**Low**	**Low**	**Very low**
2	Estimates based on self-reported energy use	**Medium**	**Low**	**Low**	**Low**	**Low**
3	Estimates based on household-level meter readings	**Medium**	**Medium**	**Medium**	**High**	**Very high**
4	Measures of real-time energy usage from smart meters	**Very low**	**Very high**	**Very high**	**Very high**	**Very high**

Note: Ratings include very low, low, medium, high and very high. The values shown in the table reflect the authors’ own subjective assessment of these criteria.

## Appendix

**Table A1.**

	Author, Year	Focuses on residential sector	Measures perceptions by appliance	Measures actual use	Included
1	Abrahamse *et al* (2007)	X	X	X	X
2	Abrahamse and Steg (2009)	X	X	X	X
3	Allcott (2011)			X	
4	Allcott (2011)	X		X	
5	Attari *et al* (2010)	X	X	X	X
6	Attari (2014).	X		X	
7	Baird and Brier (1981)	X	X	X	X
8	Barreto *et al* (2011)	X			
9	Becken (2013)			X	
10	Becker *et al* (1979)	X	X	X	X
11	Chen *et al* (2015)	X	X	X	X
12	Easton and Smith (2010)	X	X	X	X
13	Frederick *et al* (2011)	X	X	X	X
14	Gatersleben *et al* (2002)	X	X	X	X
15	Heberlein and Warriner (1983)	X		X	
16	Hirst *et al* (1982)	X		X	
17	Hirst *et al* (1987)	X		X	
18	Hori *et al* (2013)	X		X	
19	Kempton and Montgomery (1982)	X	X	X	X
20	Kempton *et al* (1985)	X	X	X	X
21	Kempton (1986)	X	X	X	X
22	Larrick and Soll (2008)			X	
23	Longstreth and Topliff (1990)	X		X	
24	Macey (1991)	X		X	
25	Meier and Deumling (2013)	X		X	
26	Mettler-Meibom and Wichmann (1982)	X	X	X	X
27	Midden *et al* (1983)	X		X	
28	Paetz *et al* 2012	X			
29	Palmborg (1986)	X		X	
30	Poortinga *et al*(2003)	X		X	
31	Raaij and Verhallen (1983)	X		X	
32	Schley and DeKay (2015)	X	X	X	X
33	Seligman *et al* (1978)	X		X	
34	Seligman *et al* (1979)	X		X	
35	Verhallen and van Raaij (1981)	X		X	
36	Wilhite and Ling (1995)	X		X	
37	Wolvén (1991)	X		X	
38	Xiaohua and Zhenming (1996)	X		X	
39	Yohanis *et al* (2008)	X		X	
